# Supplementary material for: Systematic single-variant and gene-based association testing of thousands of phenotypes in 394,841 UK Biobank exomes
Source: Cell Genom. 2022 Aug 15;2(9):100168. doi: 10.1016/j.xgen.2022.100168 (PMC9903662; doi:10.1016/j.xgen.2022.100168)
Supplement: Document S1. Figures S1–S27 and Tables S1–S6 [file mmc1.pdf]

Cell Genomics, Volume *2*

Supplemental information

Systematic single-variant and gene-based association testing of thousands of phenotypes in 394,841 UK Biobank exomes

Konrad J. Karczewski, Matthew Solomonson, Katherine R. Chao, Julia K. Goodrich, Grace Tiao, Wenhan Lu, Bridget M. Riley-Gillis, Ellen A. Tsai, Hye In Kim, Xiuwen Zheng, Fedik Rahimov, Sahar Esmaeeli, A. Jason Grundstad, Mark Reppell, Jeff Waring, Howard Jacob, David Sexton, Paola G. Bronson, Xing Chen, Xinli Hu, Jacqueline I. Goldstein, Daniel King, Christopher Vittal, Timothy Poterba, Duncan S. Palmer, Claire Churchhouse, Daniel P. Howrigan, Wei Zhou, Nicholas A. Watts, Kevin Nguyen, Huy Nguyen, Cara Mason, Christopher Farnham, Charlotte Tolonen, Laura D. Gauthier, Namrata Gupta, Daniel G. MacArthur, Heidi L. Rehm, Cotton Seed, Anthony A. Philippakis, Mark J. Daly, J. Wade Davis, Heiko Runz, Melissa R. Miller, and Benjamin M. Neale

a.

Sample 1 GVCF

CHR	POS	Ref	Alt	Sample1
chr1	3330	C	*<NON_REF>*	GT=0/0, DP=10, GQ=30, END=3330
chr1	3331	T	G, *<NON_REF>*	GT=0/1, DP=10, GQ=99, AD=[6, 4], PL=[100, 0, 105]
chr1	3332	C	*<NON_REF>*	GT=0/0, DP=11, GQ=30, END=3349
chr1	3350	A	C, *<NON_REF>*	GT=1/1, DP=15, GQ=55, AD=[0, 15], PL=[1002, 55, 0]

b.

Sample 2 GVCF

CHR	POS	Ref	Alt	Sample2
chr1	3330	C	*<NON_REF>*	GT=0/0, DP=9, GQ=30, END=3334
chr1	3335	G	C, *<NON_REF>*	GT=1/1, DP=7, GQ=22 AD=[0, 7], PL=[154, 22, 0]
chr1	3336	T	*<NON_REF>*	GT=0/0, DP=10, GQ=30, END=3349
chr1	3350	A	T, *<NON_REF>*	GT=0/1, DP=12, GQ=99 AD=[7, 5], PL=[154, 0, 102]

Merged SVCR

c.

CHR	POS	Ref	Alt	Sample1	Sample2
chr1	3330	C	*<NON_REF>*	LGT=0/0, DP=10, GQ=30, END=3330	LGT=0/0, DP=9, GQ=30, END=3334
chr1	3331	T	G, *<NON_REF>*	LA=[0, 1], LGT=0/1, DP=10, GQ=99, LAD=[6, 4], LPL=[100, 0, 105]	
chr1	3332	C	*<NON_REF>*	LGT=0/0, DP=11, GQ=30, END=3349	
chr1	3335	G	C, *<NON_REF>*		LA=[0, 1], LGT=1/1, DP=7, GQ=22 LAD=[0, 7], LPL=[154, 22, 0]
chr1	3336	T	*<NON_REF>*		LGT=0/0, DP=10, GQ=30, END=3349
chr1	3350	A	C, T, *<NON_REF>*	LA=[0, 1], LGT=1/1, DP=15, GQ=55, LAD=[0, 15], LPL=[1002, 55, 0]	LA=[0, 2], LGT=0/1, DP=12, GQ=99 LAD=[7, 5], LPL=[154, 0, 102]

Fig. S1 | Scalable Variant Call Representation (SVCR) created from two gVCF inputs. Panels a and b display information contained in gVCFs for two distinct samples in a small genomic window. Panel c represents the merged SVCR, which contains all loci present in either a or b. There is no entry for Sample2 at chr1:3331 because Sample2's gVCF does not contain the locus chr1:3331. The GT field has been renamed to LGT (local GT), and the LA (local alleles) field has been added to record the original alleles in each gVCF, which is important at chr1:3350, a locus where both input samples have a variant call. Related to STAR Methods.

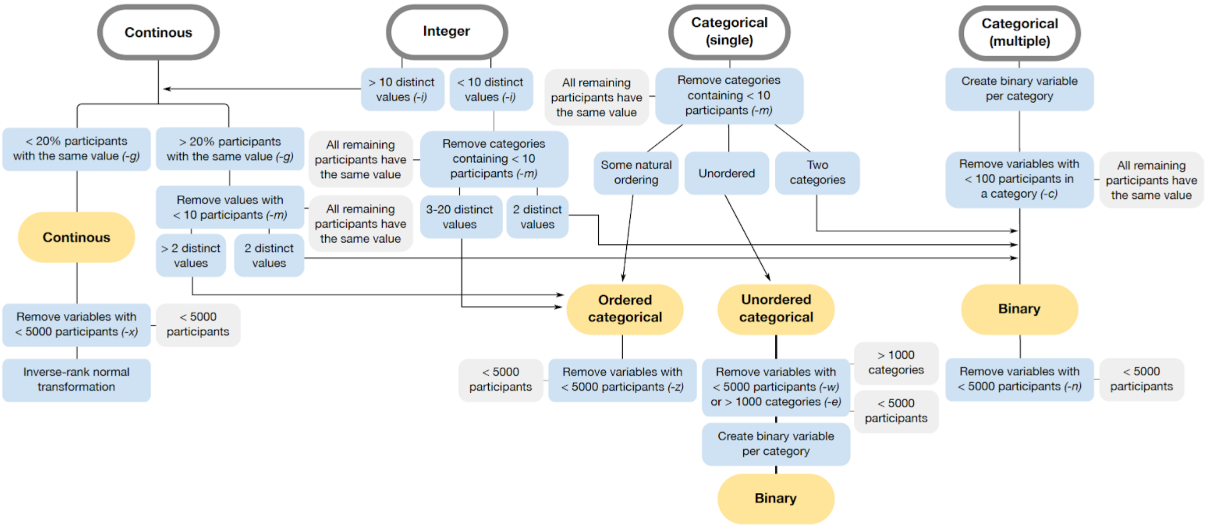

Fig. S2 | Phenotype curation pipeline. Raw phenotype data (gray outlined boxes) are passed to PHESANT, and a collection of filters (blue boxes) are applied. The thresholds shown here are the defaults in our modified version of PHESANT that can be altered in our code as desired using the flags displayed in parentheses. Grey filled boxes display the criteria for removal, and yellow filled boxes show the category of the variable after the rules in the blue boxes have been enforced. Related to STAR Methods.

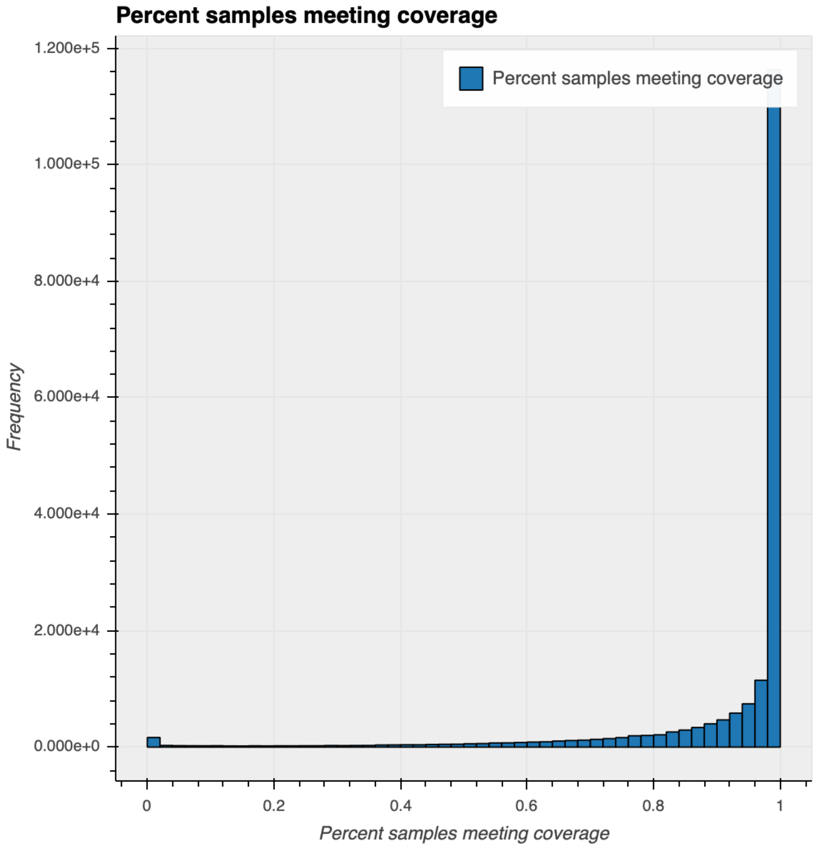

Fig. S3 | Histogram showing the percentage of samples meeting 20X mean coverage for each exome capture interval. Related to STAR Methods.

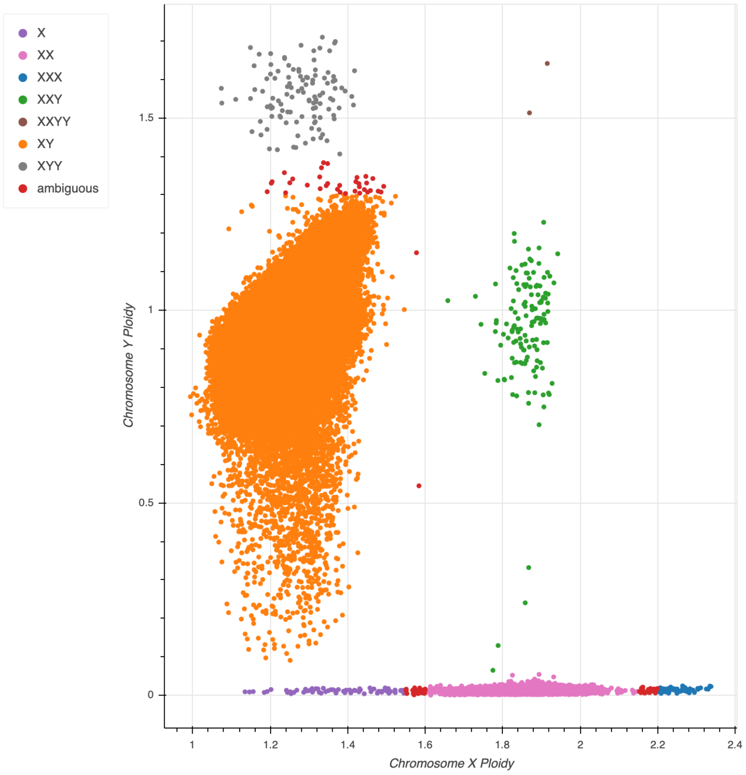

Fig. S4 | Normalized chromosome X ploidy plotted against normalized chromosome Y ploidy and colored by sex karyotype. XY samples are spread out in terms of their normalized chromosome Y coverage. This long tail of samples is likely due to mosaic loss of chromosome Y. Related to STAR Methods.

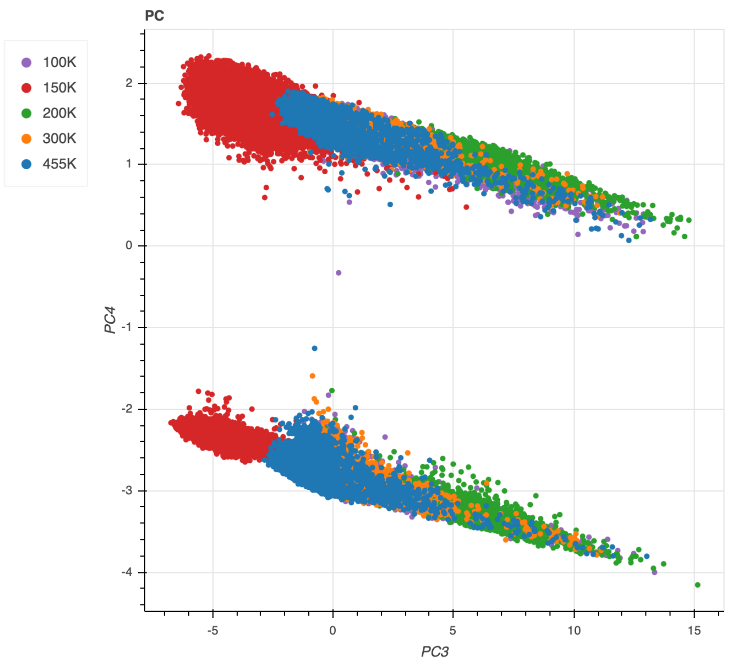

Fig. S5 | Platform inference using missingness PCA. PC3 vs PC4 colored by batch. Note that the batch names indicate the additional samples added from that batch. Thus, ‘100K’ refers to data tranche 1, ‘150K’ refers to samples added in tranche 1.5 (the first 50K samples released to the public), ‘200K’ refers to samples added in tranche 2, ‘300K’ refers to samples added in tranche 3, and '455K' refers to samples added in tranche 4. The separation in PC4 is driven by a common copy number variant. Related to STAR Methods.

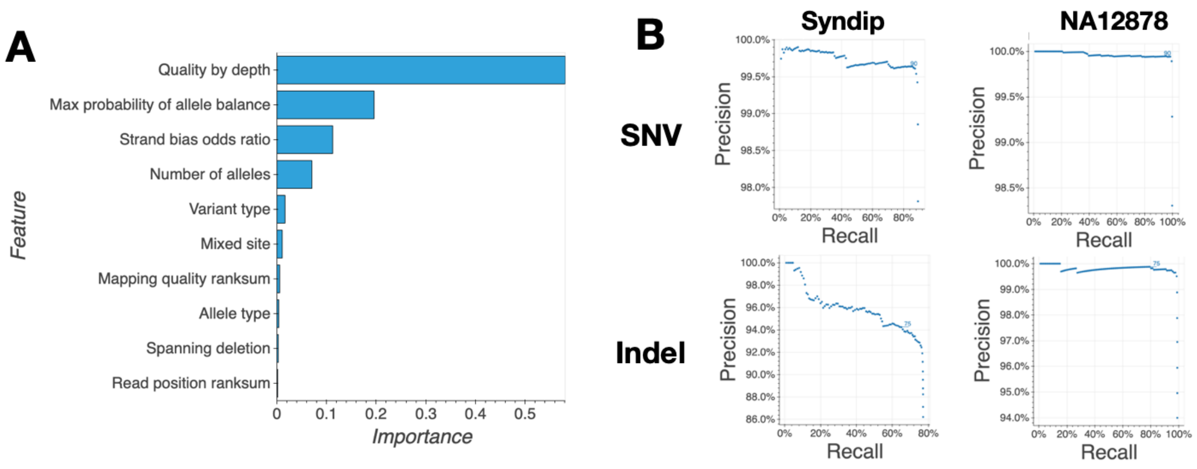

Fig. S6 | Variant QC (**A**): A summary of the features used in the random forests model and their relative importance in the model generated. (**B**): Precision and recall curves for the random forest classifier using two truth samples present in our data (NA12878 and syndip). The highlighted points at 90 for SNVs and 75 for indels indicate the cutoffs used for variant filtering. Related to STAR Methods.

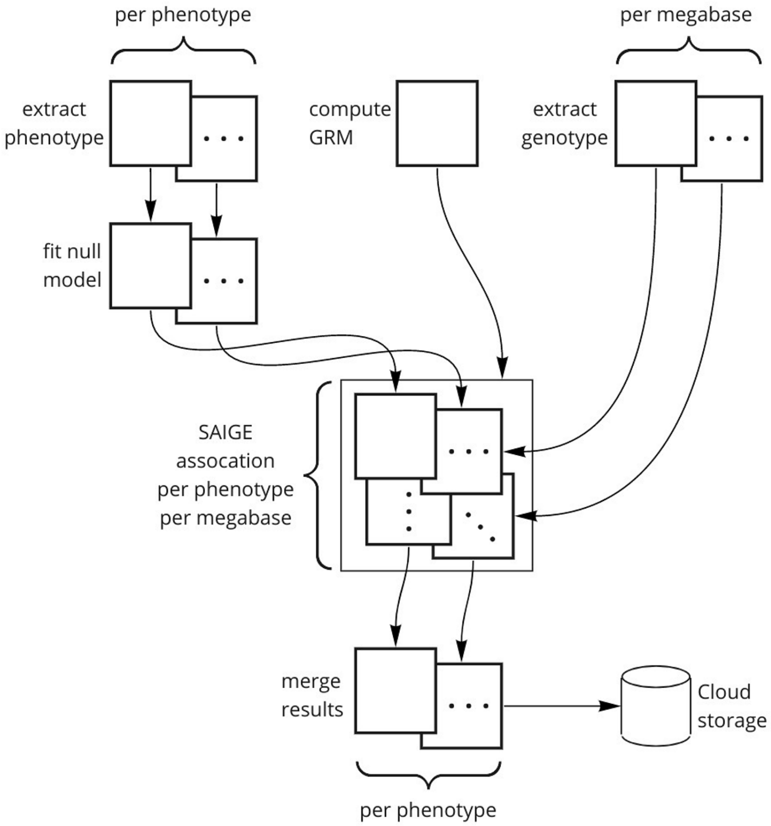

Fig. S7 | Hail Batch schematic for SAIGE association analysis. An example batch (the SAIGE pipeline used in this manuscript) is shown here. Related to STAR Methods.

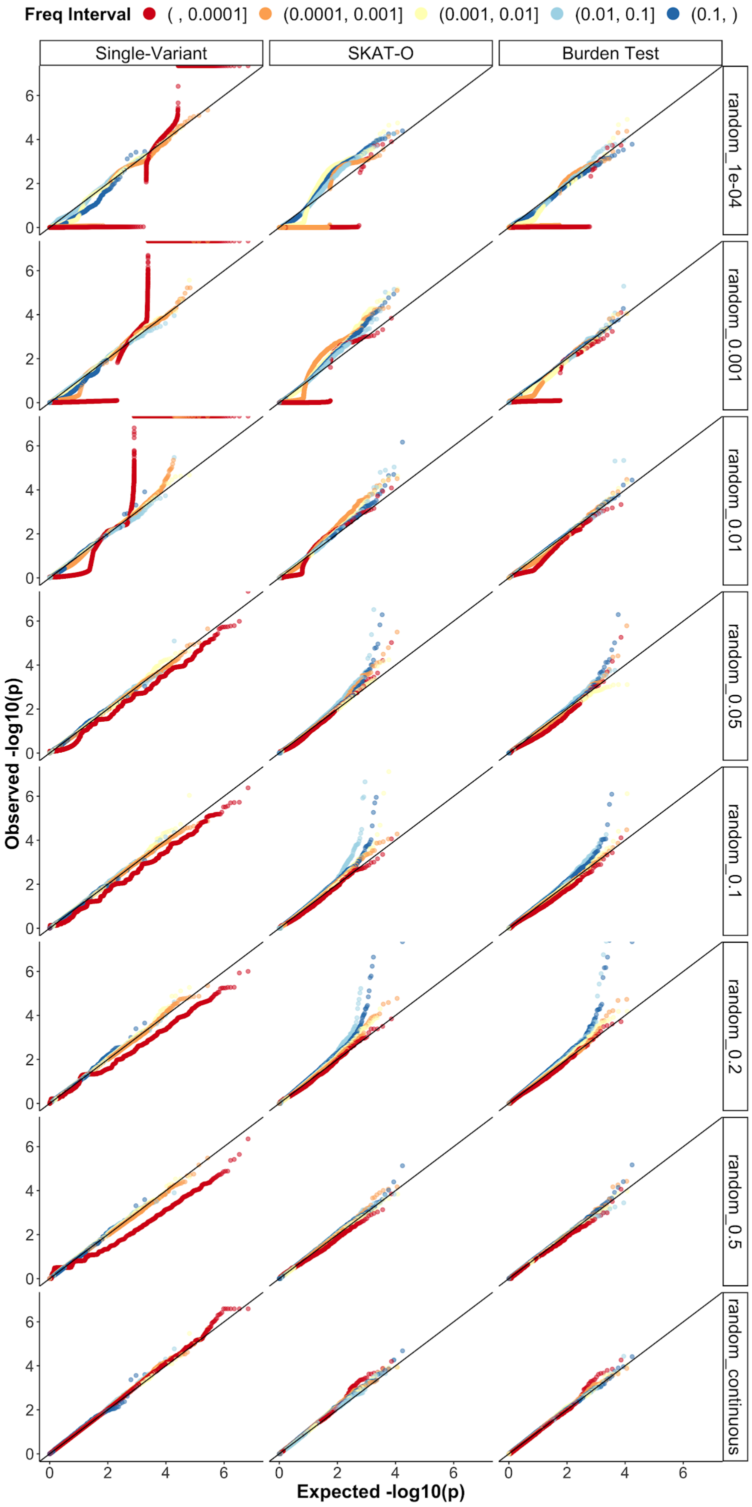

Fig. S8 | QQ-plots of randomly generated heritable (heritability = 100%) phenotypes for single-variant tests (left) and for group tests (SKAT-O, middle; and burden tests, right). The increasing prevalence of each binary phenotype is indicated by the label on the right (1e-4 to 0.5), followed by continuous traits. Related to STAR Methods.

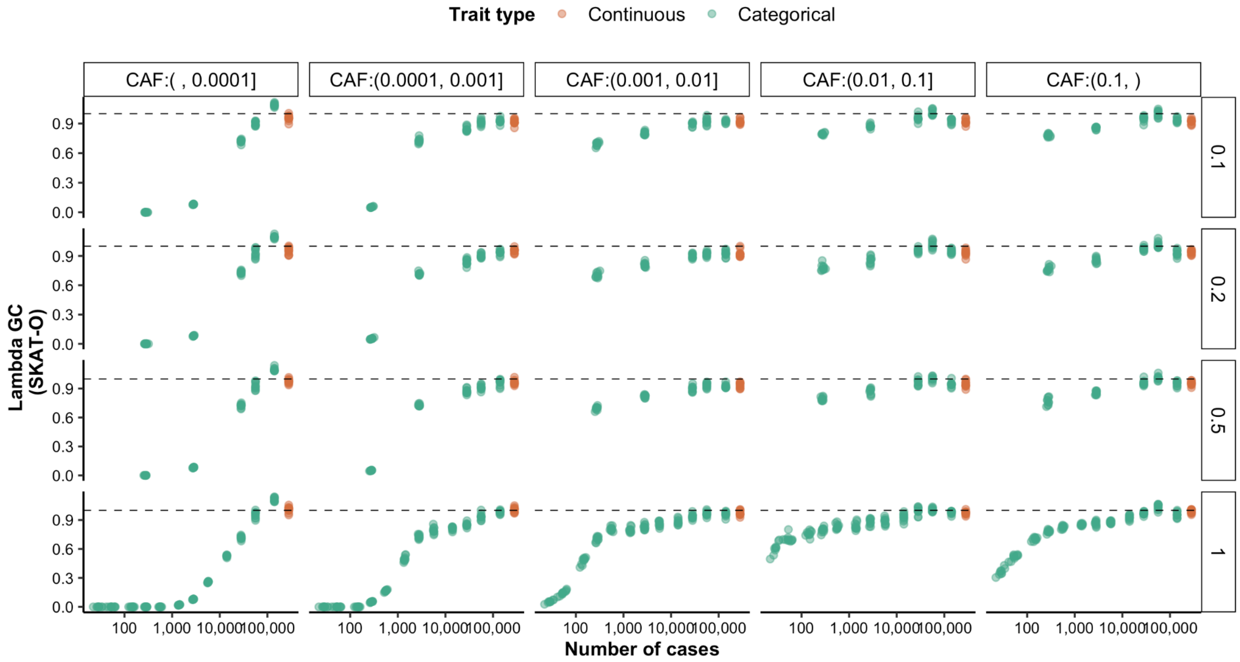

Fig. S9 | Lambda GC by cumulative allele frequency (CAF) by heritability. The heritability of the phenotypes are shown by the label on the right. Related to STAR Methods.

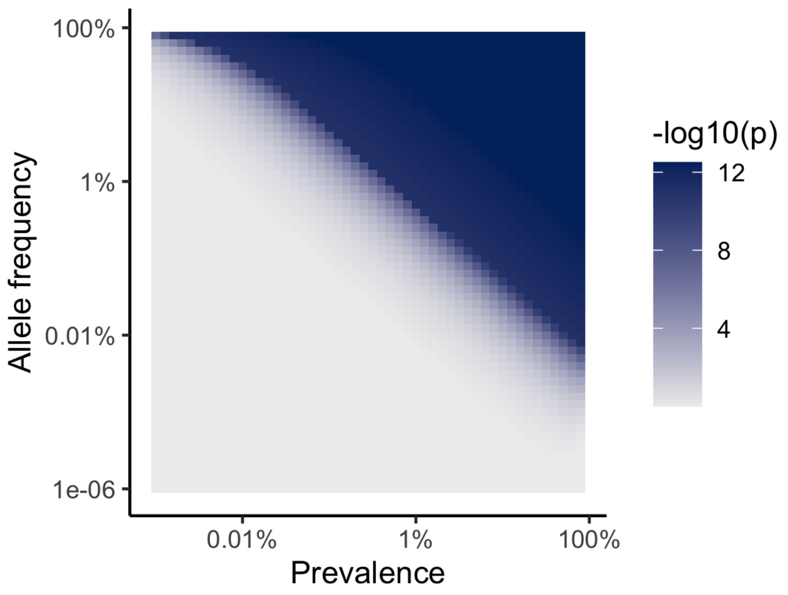

Fig. S10 | Power for rare variant associations. The minimum p-value possible from a protective mechanism with an odds ratio = 0: here, we compute the p-value of a chi-squared test of the case where the variant is absent from cases, while controls have a frequency as plotted. For the color-scale, a second logarithm is applied to p values below 10^{-10}. Related to STAR Methods.

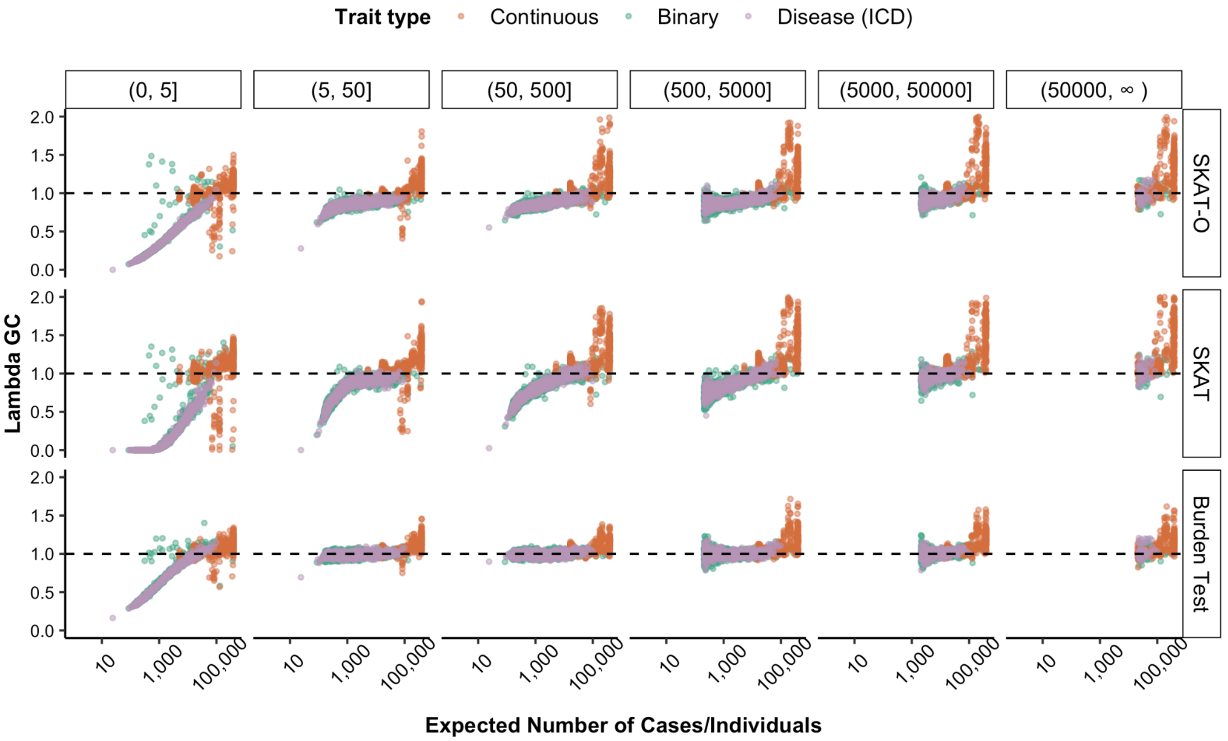

Fig. S11 | Lambda GC for each phenotype vs case count, split by expected AC interval for SKAT-O, SKAT and burden tests. Related to STAR Methods.

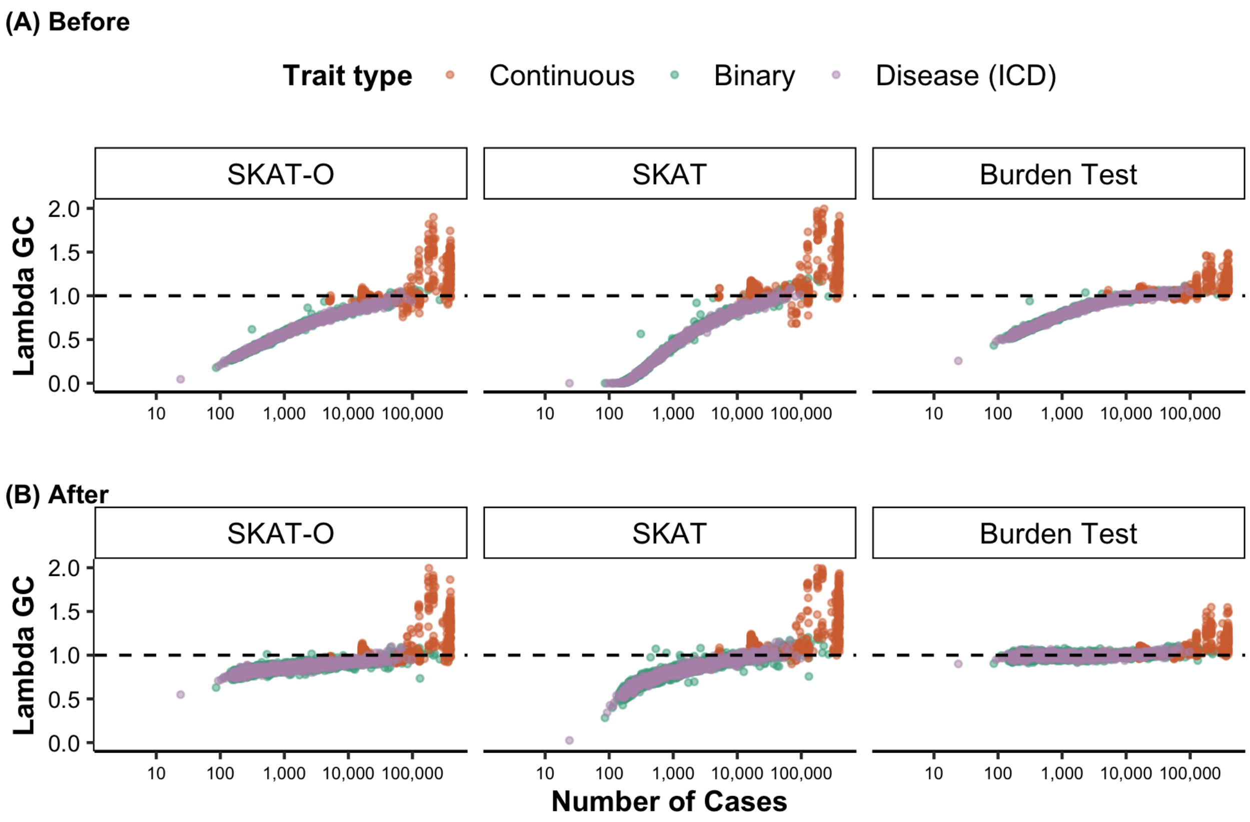

Fig. S12 | Lambda GC for each phenotype vs case count for SKAT-O, SKAT and burden tests, before and after filtering out summary statistics with expected AC < 50, number of variants tested < 2, and coverage < 20. Related to STAR Methods.

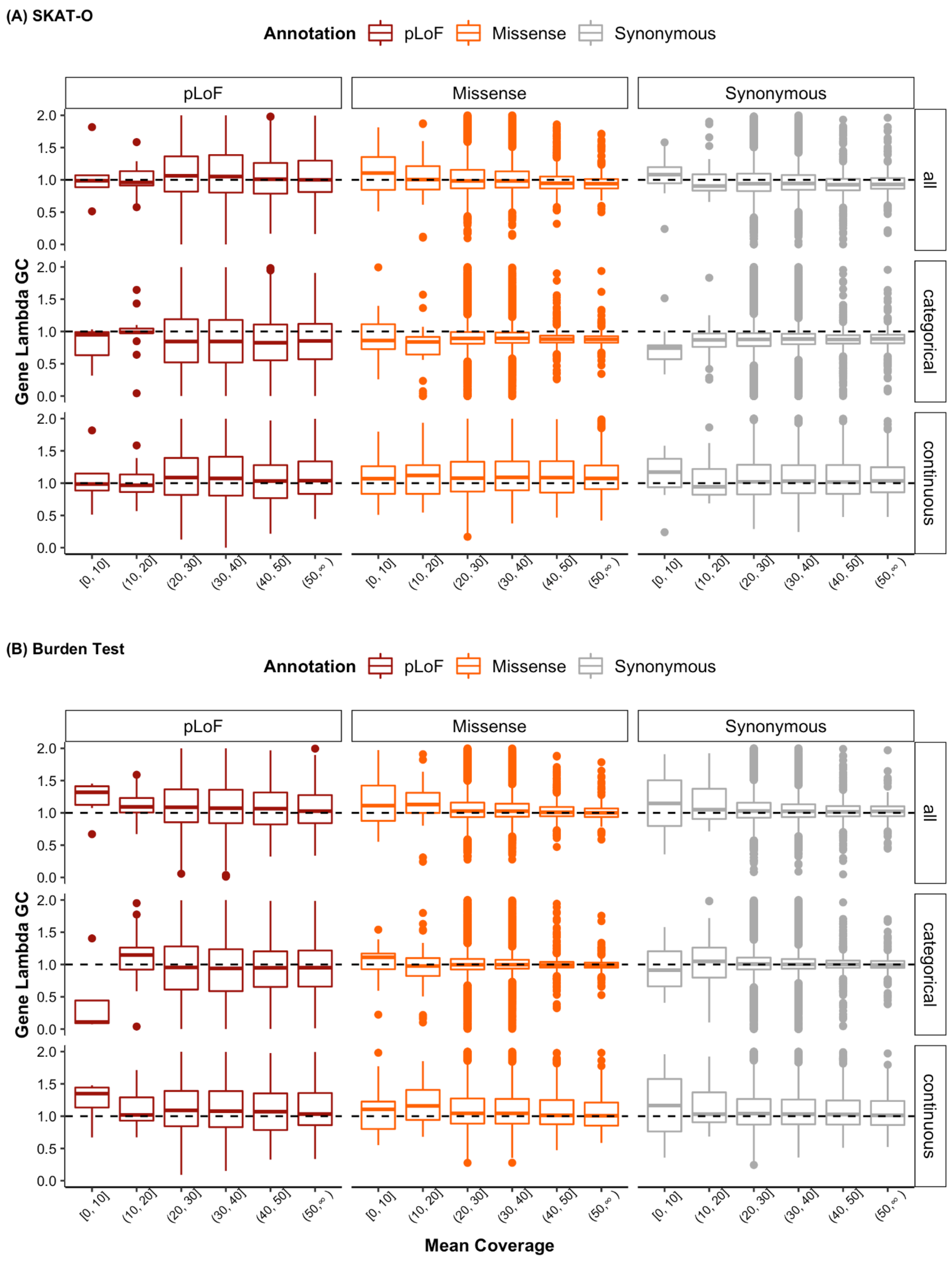

Fig. S13 | Coverage vs gene-based lambda GC, for SKAT-O (**A**) and burden tests (**B**). Related to STAR Methods.

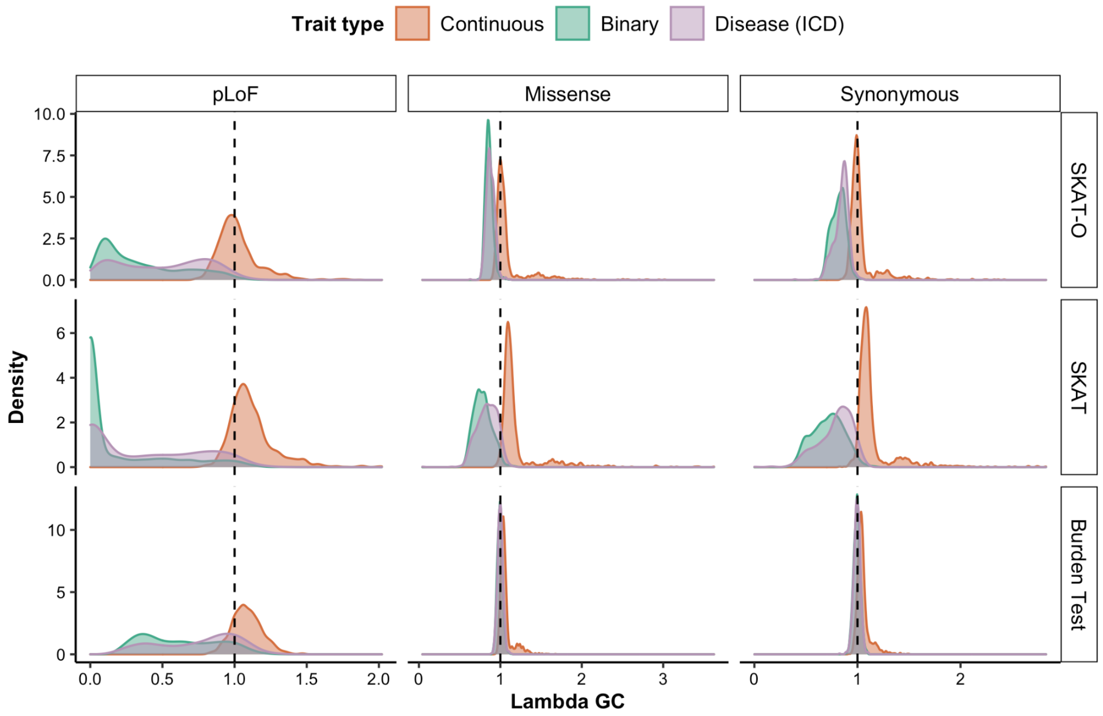

Fig. S14 | Lambda GC for each phenotype. A density plot of the distribution of lambda GC values for each phenotype is shown, broken down by trait type, test type, and set of variants used in the lambda calculation. Related to STAR Methods.

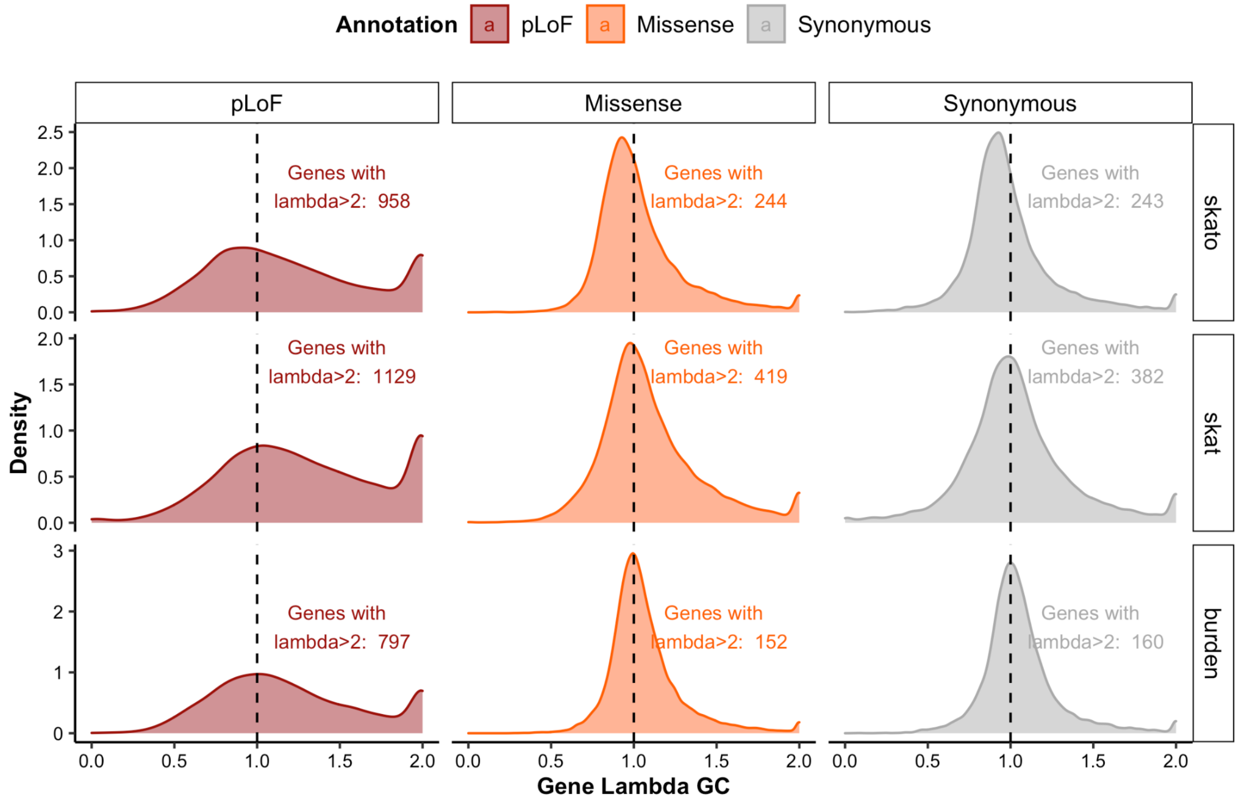

Fig. S15 | Lambda GC for each gene. A density plot of the distribution of lambda GC values for each gene is shown, broken down by test type and set of variants used in the lambda calculation. Related to STAR Methods.

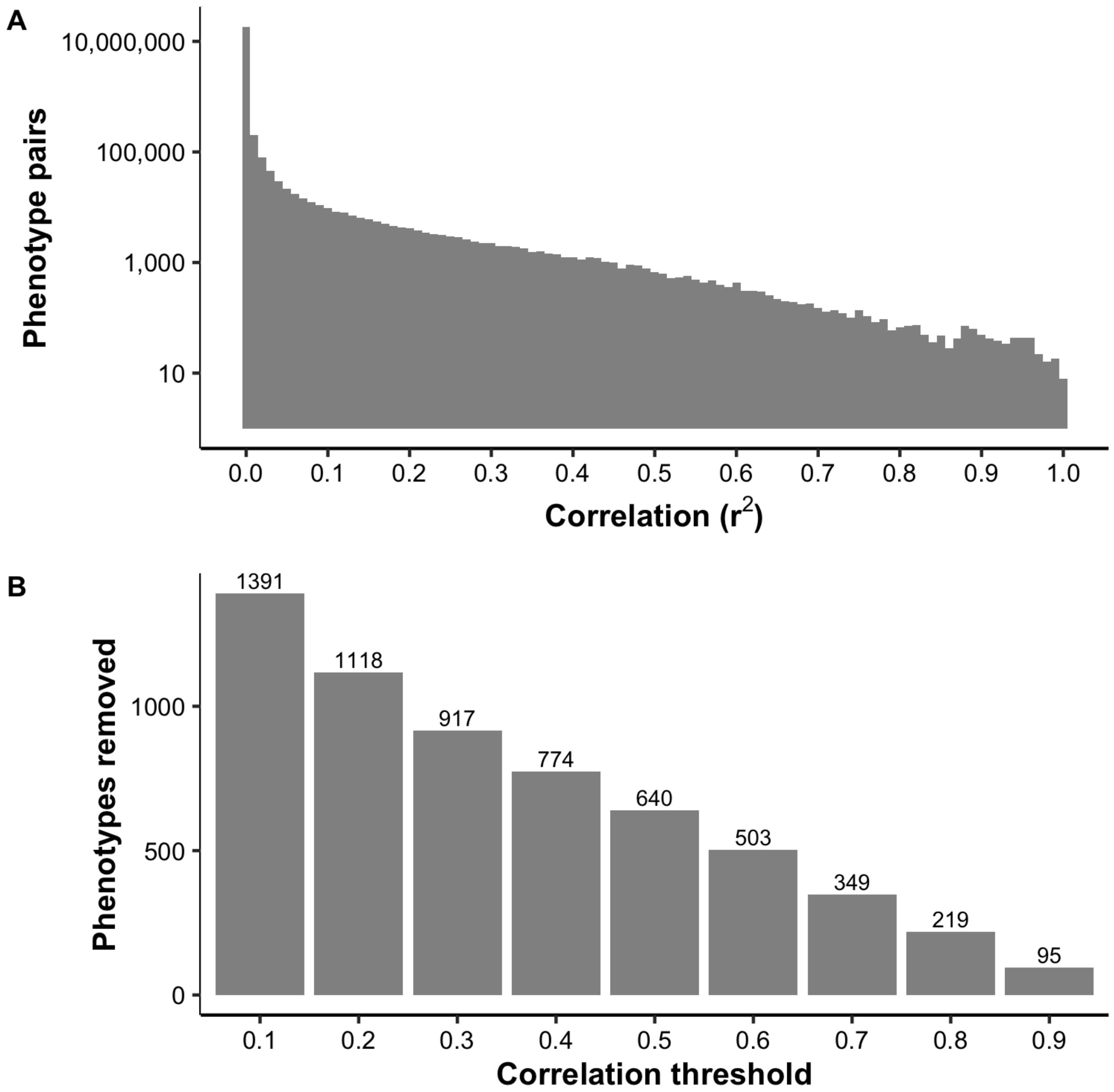

Fig. S16 | Independence of phenotypes. (**A**): A histogram of the number of phenotype pairs by correlation (r^2). (**B**): The number of phenotypes that would be removed by the maximum independent set method, by r^2 threshold. Related to STAR Methods.

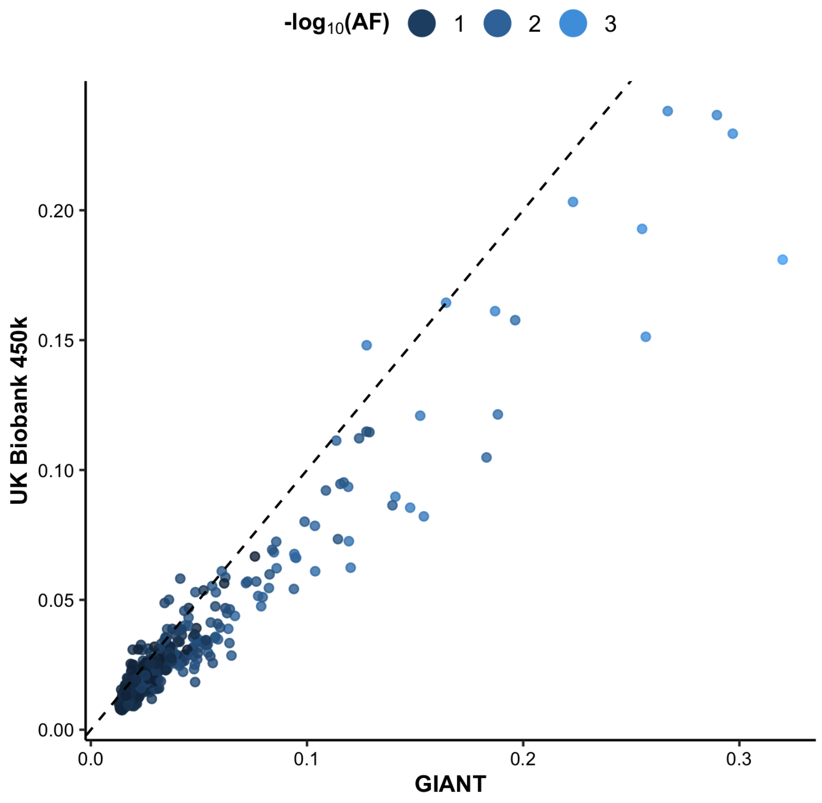

Fig. S17 | Comparison of effect sizes between UK Biobank and GIANT for height. The y = x line is shown for reference. Related to STAR Methods.

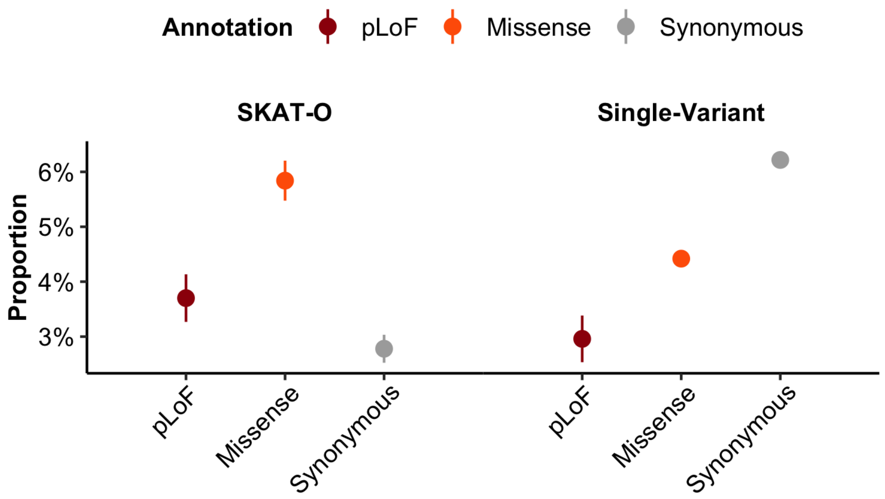

Fig. S18 | The proportion of genes (**A**) and variants (**B**) associated with at least one trait, broken down by functional class. Related to Figure 3 and STAR Methods.

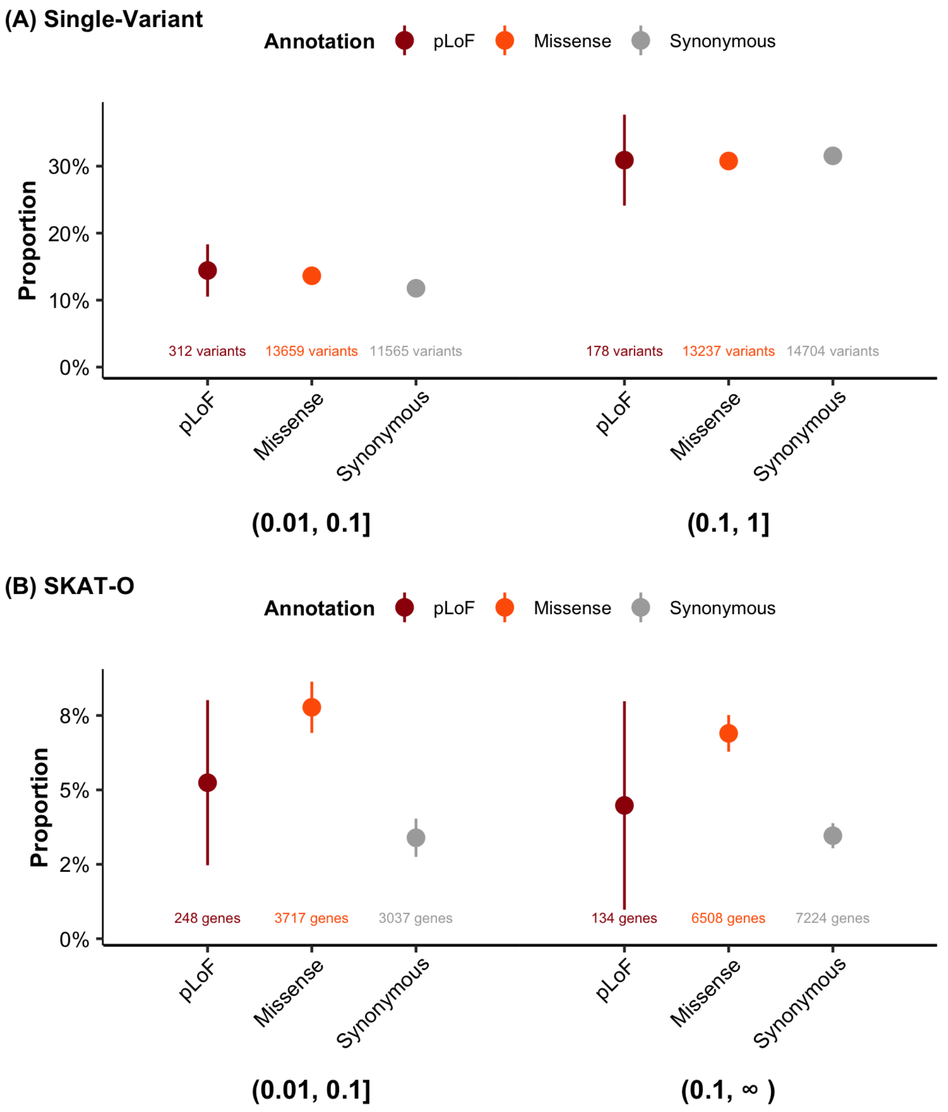

Fig. S19 | The proportion of variants (**A**) and genes (**B**) with at least one phenotype reaching our p-value threshold is shown broken down by allele frequency category (**A**) or cumulative allele frequency category (**B**) and by functional category. For common variants, missense variants show a higher proportion of associations than synonymous variants, but pLoF variants do not show a higher proportion as might be expected, likely due to artifacts at common pLoF variants. Related to Figure 3 and STAR Methods.

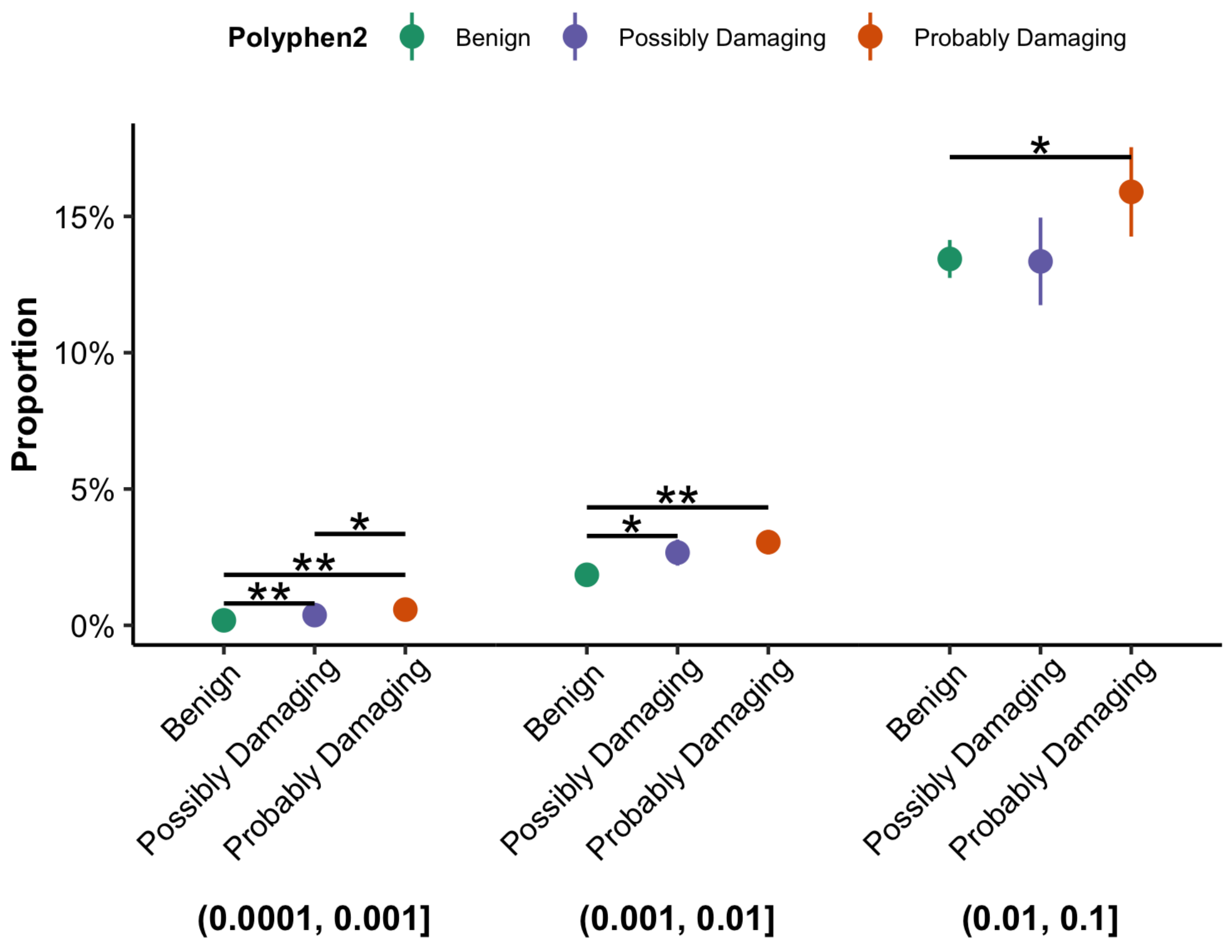

Fig. S20 | The proportion of variants with at least one association is shown broken down by PolyPhen2 annotation group and allele frequency category. * and ** indicate a significant group difference by chi-square test at $p < 0.05$ and $p < 0.001$, respectively. No significant difference is observed for allele frequencies above 10%. Related to Figure 3 and STAR Methods.

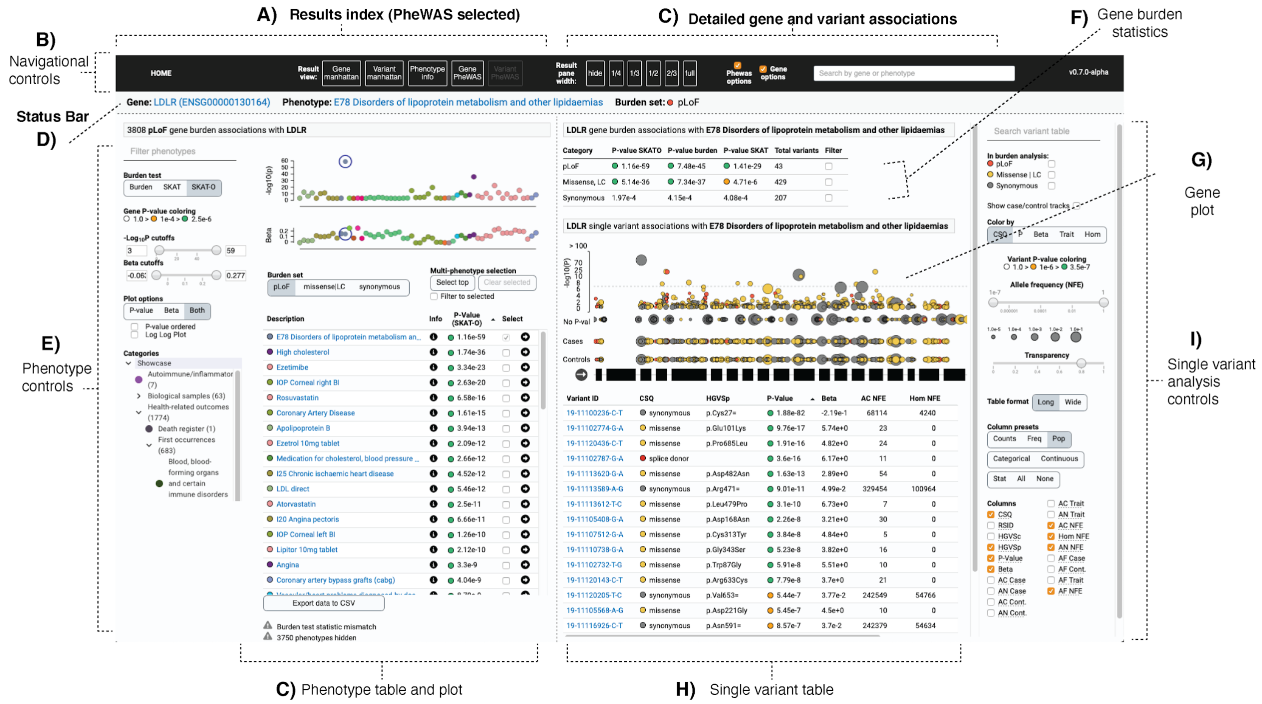

Fig. S21 | Overview of the UKBB exome gene browser interface. The left hand side of the page provides access to all associations with a given gene, variant, or phenotype. The right hand side is for exploring detailed gene test associations (burden, SKAT-O, SKAT) across annotation groups (pLoF, missense and low confidence pLoF, synonymous) in addition to single variants that were included in the burden tests. Related to STAR Methods.

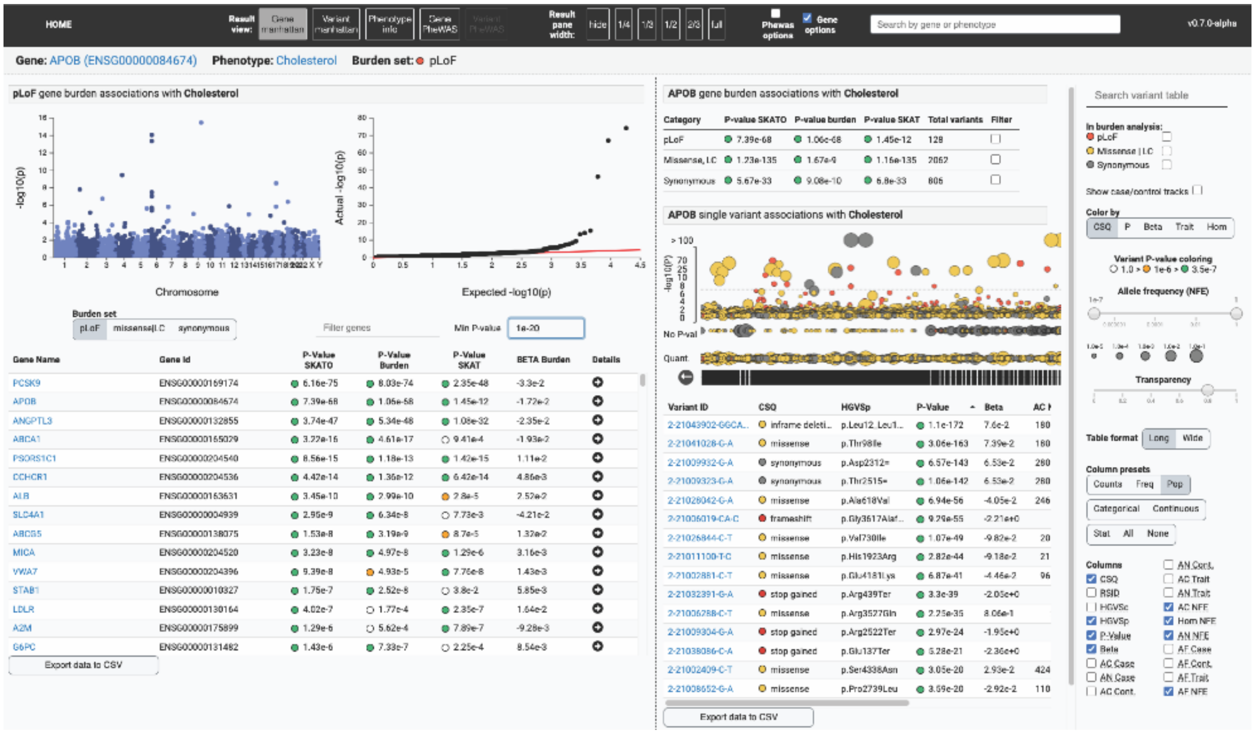

Fig. S22 | Results by phenotype. For a given phenotype, gene or variant association results are displayed in Manhattan plot formats in addition to an exportable table. Detailed gene results can be quickly previewed using the arrow button located in each row of the table. Related to STAR Methods.

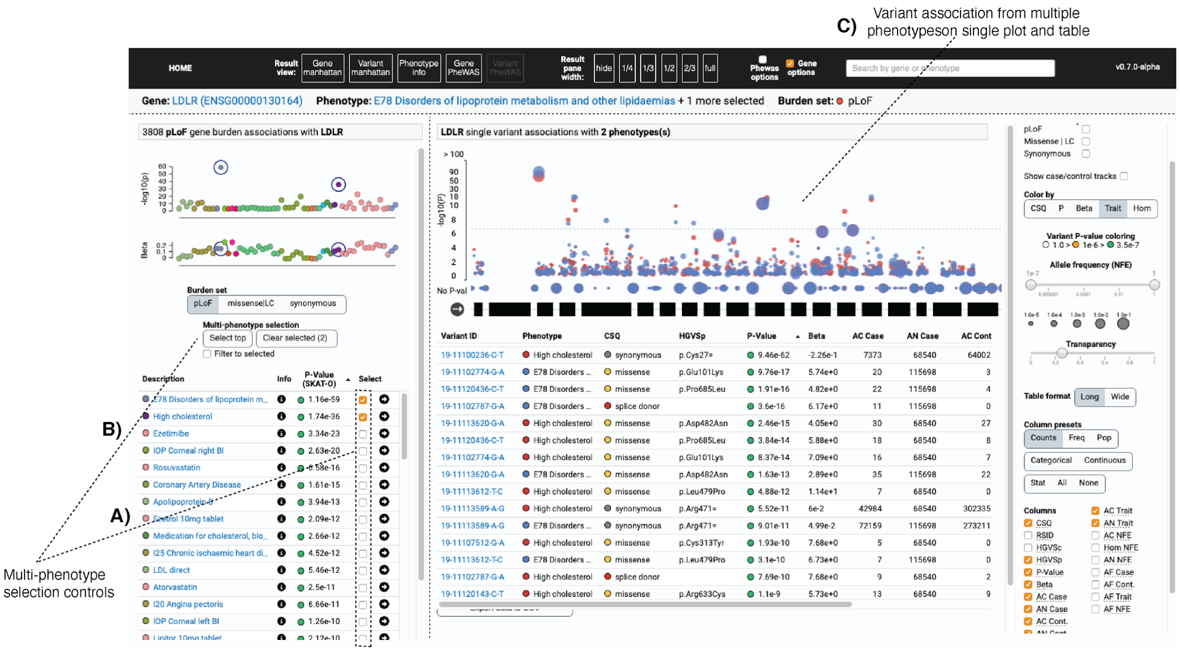

Fig. S23 | Multi-phenotype plotting. Many phenotypes can be selected simultaneously to be overlaid for comparison of single variant analysis associations. Related to STAR Methods.

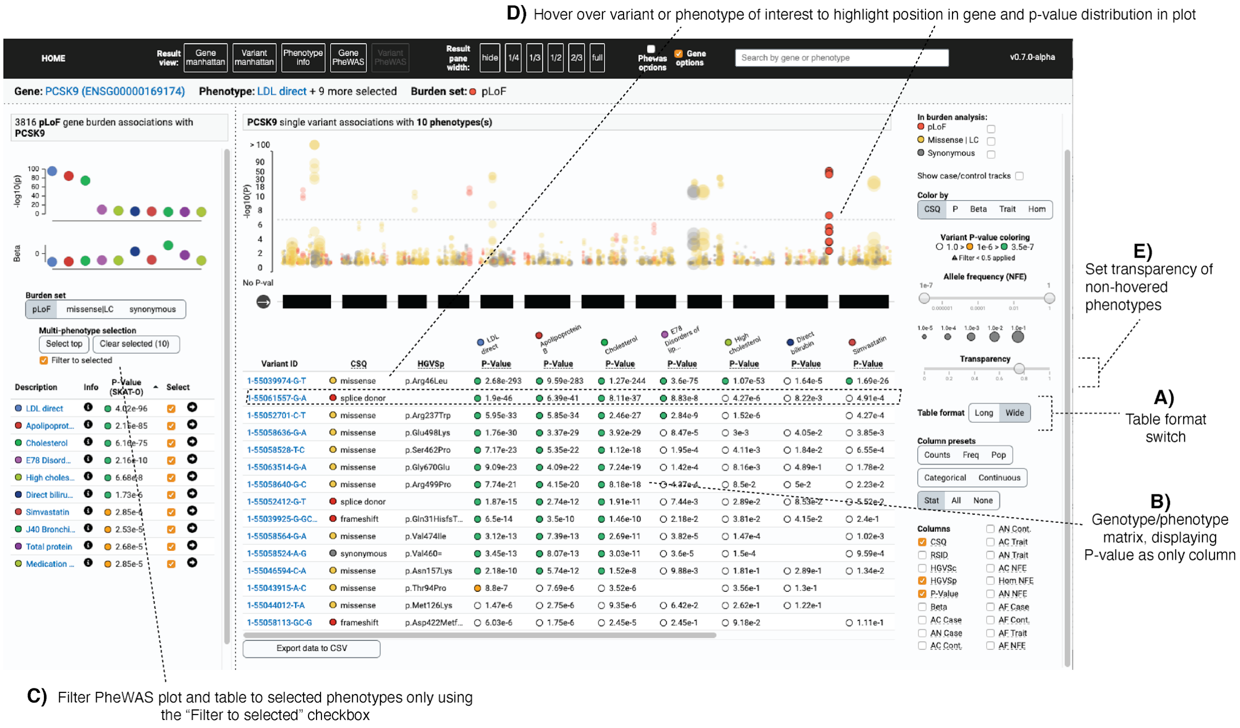

Fig. S24 | Using hover interactions with the multi-phenotype pivot table. Here 10 LDLR associations are compared simultaneously and one splice donor of interest is hovered in the variant table to highlight the plot. Related to STAR Methods.

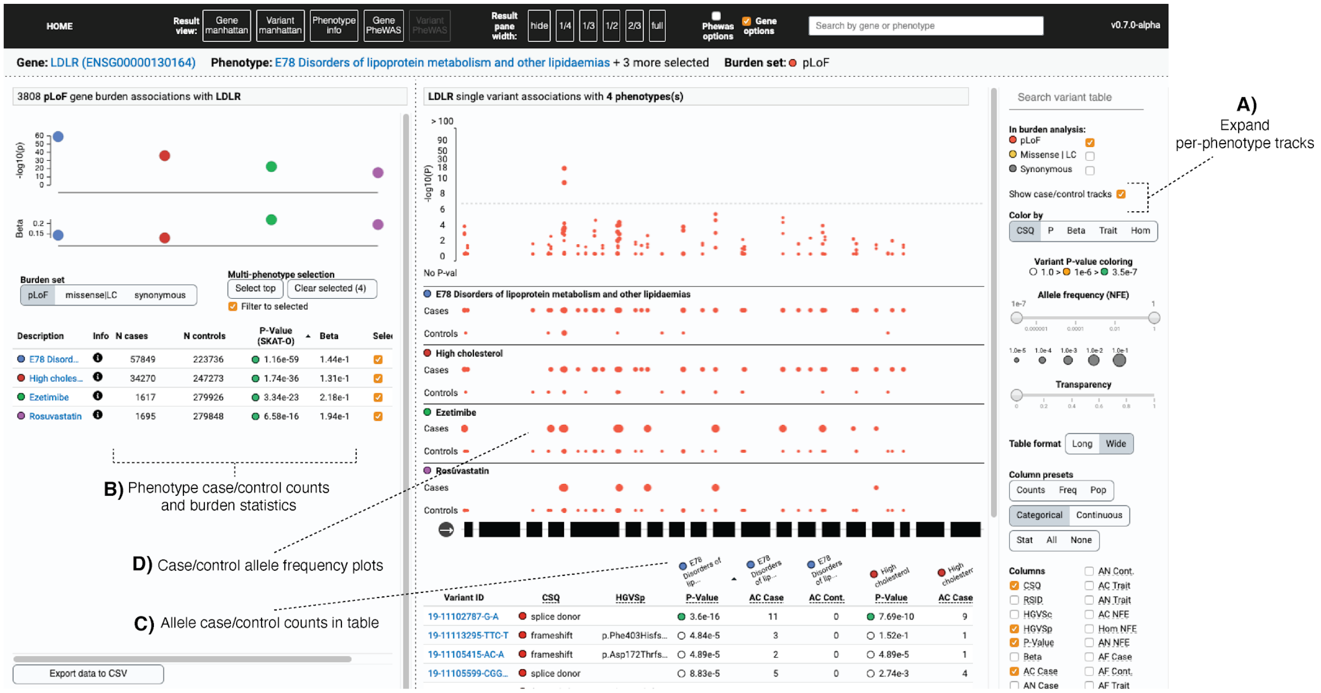

Description	Info	N cases	N controls	P-Value (SKAT-O)	Beta	Select
E78 Disord...		57849	223736	1.16e-59	1.44e-1	
High choles...		34270	247273	1.74e-36	1.31e-1	
Ezetimibe		1617	279926	3.34e-23	2.18e-1	
Rosuvastatin		1695	279848	6.58e-16	1.94e-1	

Variant ID	CSQ	HGVSp	P-Value	AC Case	AC Cont.	P-Value	AC Case
19-11102787-G-A	splice donor		3.6e-16	11	0	7.69e-10	9
19-11113295-TTC-T	frameshift	p.Phe403Hisfs...	4.84e-5	3	0	1.52e-1	1
19-11105415-AC-A	frameshift	p.Asp172Thrfs...	4.89e-5	2	0	4.89e-5	1
19-11105599-CGG...	splice donor		8.83e-5	5	0	2.74e-3	4

Fig. S25 | Viewing case-control counts and allele frequencies for pLoF variants across traits in a gene. Related to STAR Methods.

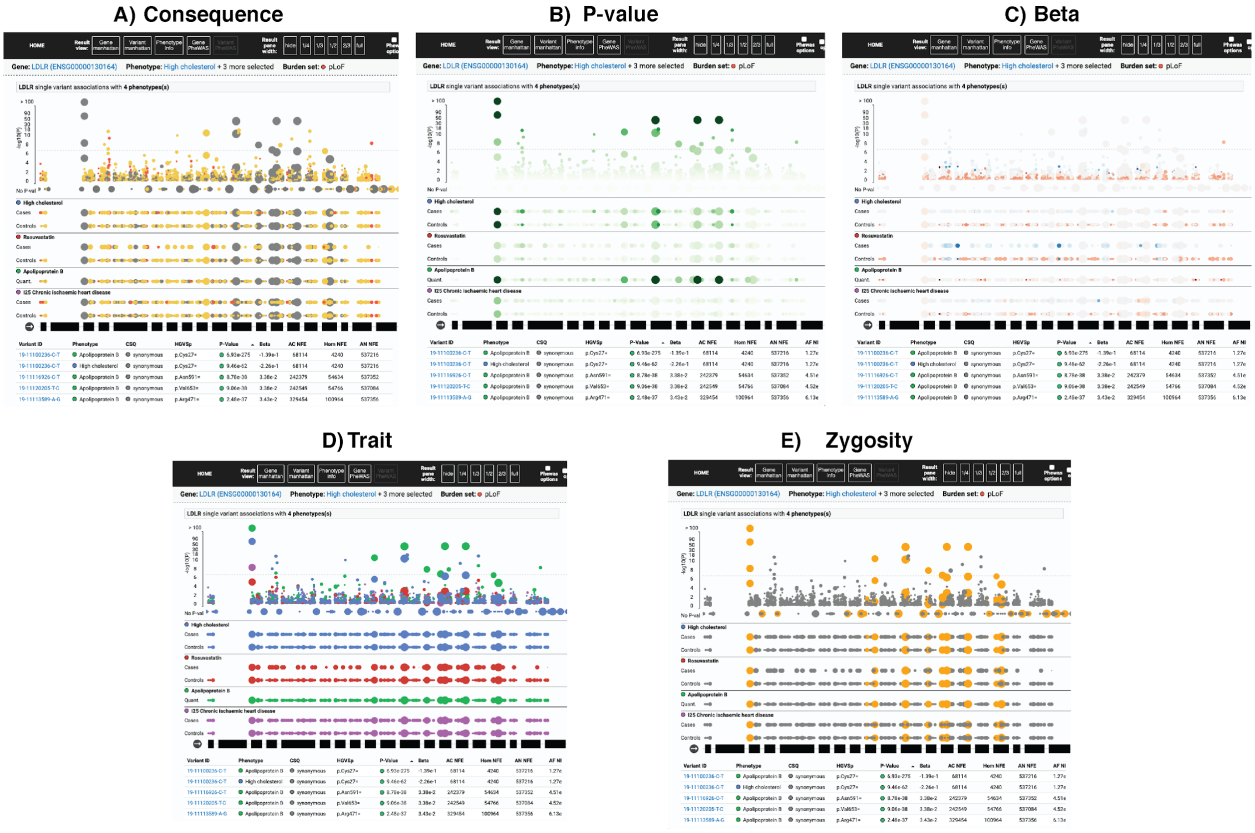

Fig. S26 | Color variants by attribute to uncover patterns in A) consequence, B) p-value, C) beta, D) trait, or E) zygosity. Related to STAR Methods.

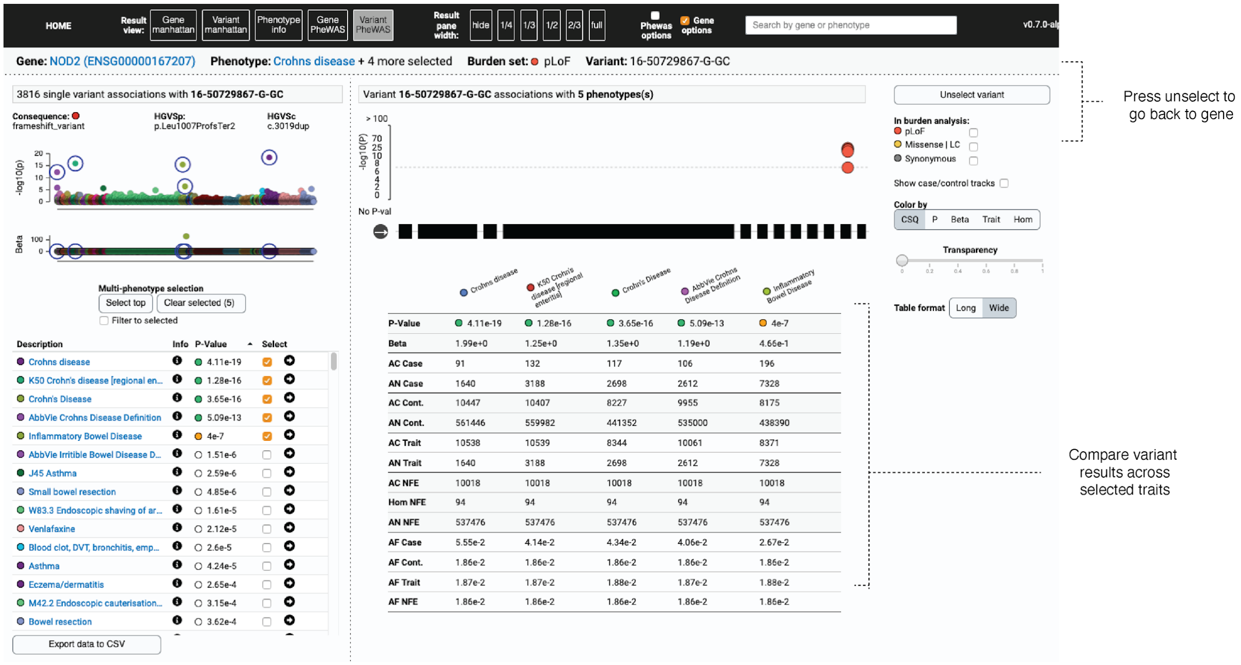

Fig. S27 | Single variant page. Related to STAR Methods.

Table S1 | Final sample counts passing QC. "nfe" refers to samples inferred as having non-Finnish European ancestry. Note that relatedness was run after hard filtering, so the total number of related and unrelated individuals is equal to the total number of samples less 683. Related to STAR Methods.

Category	Related	Unrelated	All
Total	29900	424114	454014
Hard filtered			683
Outlier filtered	184	2877	3061
High quality	29716	421237	450953
High quality (EUR)	26891	367963	394854

Table S2 | QC of summary statistics. All filters are applied sequentially. Related to STAR Methods.

	Description	Count (% Percentage Remaining)			
		**pLoF**	**Missense**	**Synonymous**	**Total**
**Group (SKAT-O)**	Before filtering	18,358	19,403	19,372	75,767 (Oth:18,634)
	Number of variants >= 2	17,876 (97.4%)	19,392 (99.9%)	19,355 (99.9%)	75,251 (99.3%) (Oth:18,628)
	Mean coverage >= 20	17,370 (94.6%)	18,791 (96.8%)	18,768 (96.9%)	72,999 (96.3%) (Oth:18,070)
	At least 1 phenotype with expected AC (CAF*n_cases) >= 50	8,044 (43.8%)	18,461 (95.1%)	18,068 (93.3%)	62,350 (82.3%) (Oth:17,777)
	Lambda of the synonymous group > 0.75	7,296 (39.7%)	15,943 (82.2%)	16,014 (82.7%)	54,647 (72.1%) (Oth:15,394)
**Variant**	Before filtering	515,246	5,279,243	2,274,565	8,074,878 (NA: 5,824)
	Annotation defined	515,246	5,279,243	2,274,565	8,069,054 (99.9%)
	At least 1 phenotype with expected AC (AF*n_cases) >= 50	6,117 (1.2%)	155,705 (2.9%)	101,874 (4.5%)	263,696 (3.3%)
		**Continuous**	**Categorical**	**Disease (ICD)**	**Total**
**Phenotype (SKAT-O)**	Before filtering	1,233	2,571	725	4,529
	Lambda > 0.75	1,233 (100%)	2,514 (97.8%)	710 (97.9%)	4,457 (98.4%)
	Correlation < 0.5	677 (54.9%)	2,434 (94.7%)	708 (97.7%)	3,819 (84.3%)

Table S3 | Comparison to 32 rare (MAF < 1%) variants associated with adult height in GIANT; in UK Biobank, 21 of these variants are found to be associated with height at $p < 8 \times 10^{-9}$ (blue), and 29 are associated with height at $p < 0.05$ (light blue). Related to STAR Methods.

Locus	Allele (Ref)	Allele (Alt)	Annotation	Gene	P-value			
					UK Biobank	GIANT		
						Discovery	Validation	Combined
1:32673514	G	C	missense	IQCC	1.11E-10	7.92E-08	3.83E-06	1.34E-12
1:41540902	G	A	missense	SCMH1	1.34E-27	1.58E-25	9.42E-13	1.35E-36
1:41618297	G	A	missense	SCMH1	6.88E-24	1.92E-15	1.32E-08	1.80E-22
1:149902342	C	T	missense	MTMR11	1.82E-14	4.16E-06	7.11E-06	3.03E-10
1:183495812	A	G	missense	SMG7	5.47E-14	4.97E-11	8.94E-05	1.61E-14
1:223178026	T	C	missense	DISP1	NA	1.11E-09	1.22E-06	1.27E-14
2:219920461	T	A	missense	IHH	1.17E-06	1.09E-15	1.48E-09	1.85E-23
2:220078652	C	T	missense	ABCB6	3.13E-16	3.43E-13	4.40E-04	2.47E-15
3:46939587	C	T	missense	PTH1R	9.93E-09	1.30E-11	5.48E-10	1.14E-19
4:73179445	C	T	missense	ADAMTS3	5.40E-10	1.82E-08	1.32E-04	1.30E-11
4:120422407	T	G	missense	PDE5A	1.04E-10	7.50E-17	1.28E-08	2.65E-23
5:32784907	G	A	missense	NPR3	3.93E-22	1.05E-08	1.78E-06	7.91E-14
5:64766798	G	A	missense	ADAMTS6	9.39E-17	7.82E-09	1.37E-08	4.80E-16
5:127668685	G	T	missense	FBN2	1.04E-30	2.47E-33	5.06E-20	1.47E-52
5:172755066	C	A	missense	STC2	2.25E-34	5.69E-15	1.32E-17	1.15E-30
6:155450779	A	G	missense	TIAM2	NA	1.45E-08	8.50E-01	3.96E-08
7:73482987	G	A	missense	ELN	1.48E-13	2.63E-06	1.51E-03	2.31E-08
8:135614553	G	C	missense	ZFAT	2.66E-45	4.42E-26	1.20E-14	6.12E-38
8:135622851	G	A	missense	ZFAT	4.76E-14	1.54E-12	5.94E-18	2.05E-28
11:27016360	G	A	missense	FIBIN	3.70E-08	5.79E-12	1.56E-03	3.26E-14
11:94533444	G	A	missense	AMOTL1	1.96E-06	9.01E-16	3.84E-07	2.84E-21
12:58138971	G	A	missense	TSPAN31	4.63E-01	8.26E-08	2.85E-03	5.50E-09
12:121756084	G	A	missense	ANAPC5	4.32E-15	1.09E-11	1.44E-11	1.45E-21
15:44153571	C	T	missense	WDR76	1.05E-04	1.56E-06	3.42E-04	2.32E-09

15:89424870	G	T	missense	HAPLN3	1.51E-33	2.84E-13	2.43E-11	1.02E-22
16:31474091	A	G	missense / splice acceptor	ARMC5	5.76E-10	5.88E-12	1.16E-03	1.62E-13
16:47684830	C	A	missense	PHKB	1.39E-06	3.96E-14	1.04E-01	3.43E-12
16:67470505	G	A	missense	HSD11B2	4.15E-08	1.27E-07	3.38E-04	1.97E-10
16:84900645	G	A	missense	CRISPLD2	5.32E-14	9.13E-12	4.34E-09	2.92E-19
16:84902472	G	A	missense	CRISPLD2	2.66E-22	7.75E-14	3.49E-08	2.36E-20
16:88798919	G	T	missense	PIEZO1	4.38E-17	5.27E-12	1.99E-08	8.68E-19
X:66941751	C	G	missense	AR	1.06E-08	7.05E-07	7.12E-09	2.67E-14

Table S4 | Comparison to 59 low-frequency (MAF between 1% and 5%) variants associated with adult height in GIANT; in UK Biobank, 10 of the variants were not tested, 30 of these variants are found to be associated with height at $p < 8 \times 10^{-9}$ (blue), and 49 are associated with height at $p < 0.05$ (light blue). Related to STAR Methods.

Locus	Allele (Ref)	Allele (Alt)	Annotation	Gene	P-value			
					UK Biobank	GIANT Discovery	GIANT Validation	GIANT Combined
1:51873967	G	A	missense	EPS15	3.84E-18	5.07E-08	7.60E-11	2.56E-17
1:119427467	A	C	missense	TBX15	6.10E-31	1.61E-24	4.19E-15	2.79E-36
1:150551327	G	A	missense	MCL1	1.33E-25	2.16E-09	7.86E-12	1.55E-19
1:154987704	C	T	missense	ZBTB7B	1.82E-12	7.30E-17	4.46E-10	3.46E-25
1:180886140	C	T	missense	KIAA1614	1.50E-05	1.41E-06	4.51E-04	2.63E-09
2:20205541	C	T	missense	MATN3	NA	2.67E-23	6.60E-19	3.74E-41
2:219949184	C	T	intron	NHEJ1	NA	5.96E-21	1.12E-15	8.20E-37
2:179474668	G	A	missense	TTN	NA	1.35E-07	2.15E-01	3.44E-07
2:233077064	A	G	intron	DIS3L2	NA	2.35E-16	2.58E-15	6.46E-31
3:14214524	G	A	missense	XPC	1.22E-09	1.22E-08	1.68E-02	1.29E-08
3:47162886	C	T	missense	SETD2	1.30E-08	2.24E-08	2.22E-07	1.65E-13
3:49162583	C	T	missense	LAMB2	2.72E-37	3.28E-12	1.33E-16	3.49E-27
3:98600385	T	C	missense	DCBLD2	4.69E-04	1.23E-07	5.62E-05	1.68E-12
4:5016883	G	A	missense	CYTL1	8.93E-18	2.01E-17	6.68E-11	1.86E-25
4:87730980	C	T	missense	PTPN13	6.71E-36	1.94E-19	1.38E-15	9.43E-32
4:135121721	T	C	missense	PABPC4L	4.83E-07	1.39E-13	1.33E-04	7.54E-16
4:144359490	C	T	missense	GAB1	8.42E-07	1.04E-08	3.24E-04	4.29E-12
4:154557616	C	T	missense	TMEM131L	4.32E-08	7.75E-08	5.75E-06	2.18E-12
5:102338811	A	G	missense	PAM	NA	3.76E-06	8.47E-06	1.63E-10
5:126250812	C	T	missense	MARCH3	5.87E-05	4.25E-08	2.45E-03	1.67E-10
5:135288632	A	G	missense	LECT2	7.90E-06	1.02E-07	4.77E-04	1.36E-09
5:172196752	A	G	missense	DUSP1	6.30E-14	4.00E-14	1.26E-06	1.93E-20
5:176637471	G	A	missense	NSD1	3.58E-23	2.38E-17	2.62E-12	4.27E-30

5:176722005	G	A	missense	NSD1	1.01E-37	1.86E-26	8.42E-18	2.32E-41
6:30851933	G	A	intron	DDRI	NA	1.11E-08	1.24E-05	4.64E-13
6:34730395	C	T	synonymous	SNRPC	5.24E-52	9.21E-33	9.59E-31	3.45E-60
6:41903798	C	A	missense	CCND3	1.74E-41	5.51E-17	3.41E-08	1.28E-22
7:99489571	G	A	3'UTR	TRIM4	NA	3.28E-10	2.26E-07	1.40E-17
7:100490077	G	A	synonymous	ACHE	3.34E-06	8.59E-10	2.92E-02	2.98E-10
7:135123060	G	C	missense	CNOT4	4.59E-20	2.31E-17	5.04E-10	3.90E-26
8:42226805	C	G	missense	POLB	8.53E-05	1.95E-06	1.30E-02	1.88E-07
9:34660864	C	T	missense	IL11RA	7.28E-11	5.20E-13	4.42E-03	4.01E-13
9:95063947	C	T	missense	NOL8	3.67E-04	2.56E-06	3.45E-02	3.33E-06
10:79580976	G	A	missense	DLG5	1.68E-21	2.72E-11	5.15E-11	7.66E-20
10:97919011	A	G	missense	ZNF518A	1.29E-05	9.94E-08	3.05E-03	3.91E-09
11:65715204	G	A	missense	TSGA10IP	2.23E-41	1.82E-21	1.41E-23	1.52E-43
12:7548996	C	G	missense	CD163L1	1.05E-03	4.11E-08	6.68E-02	1.87E-08
12:69140339	G	C	missense	SLC35E3	1.87E-10	1.13E-09	5.UE-04	1.29E-11
12:104408832	T	C	missense	GLT8D2	NA	8.72E-10	5.82E-10	1.60E-17
13:50842259	G	A	intron	DLEU1	NA	2.33E-37	7.02E-25	5.66E-57
14:23313633	G	A	missense	MMP14	5.63E-08	1.72E-08	7.81E-09	3.27E-16
14:24707479	G	A	missense	GMPR2	1.38E-16	3.67E-16	1.34E-11	2.13E-29
14:45403699	C	A	missense	KLHL28	1.53E-07	1.55E-06	4.13E-04	3.05E-09
14:70633411	C	T	missense	SLC8A3	4.05E-11	2.49E-11	2.02E-06	2.03E-16
14:94844947	C	T	missense	SERPINA1	1.53E-100	1.39E-45	2.50E-34	1.72E-75
14:101349454	G	T	missense	RTL1	7.09E-12	1.17E-11	2.12E-04	2.50E-15
15:34520687	T	C	missense	EMC4	6.45E-02	1.16E-06	2.19E-02	1.60E-07
15:72462255	C	T	missense	GRAMD2A	2.04E-27	8.72E-17	3.66E-13	1.28E-27
15:89388905	C	T	synonymous	ACAN	1.61E-150	4.30E-72	1.08E-56	3.79E-130
16:4812705	A	G	missense	ZNF500	4.21E-10	8.61E-17	2.34E-07	2.89E-21
16:24804954	A	T	missense	TNRC6A	3.87E-13	1.08E-09	1.65E-07	1.90E-15
16:67409180	G	A	missense	LRRC36	2.22E-19	1.08E-18	3.91E-13	6.40E-31

17:67081278	A	G	missense	ABCA6	5.70E-14	2.17E-06	5.58E-07	5.57E-12
18:74980601	A	T	missense	GALR1	6.28E-07	3.60E-18	3.64E-05	5.11E-19
19:45296806	C	T	missense	CBLC	5.91E-03	1.48E-07	1.19E-02	2.96E-08
19:55879672	C	T	missense	IL11	5.24E-47	1.02E-57	2.28E-23	5.32E-81
19:55993436	G	T	missense	ZNF628	9.38E-47	2.28E-18	1.17E-18	6.33E-34
22:28501414	C	T	missense	TTC28	NA	9.47E-11	3.24E-09	3.93E-19
22:42095658	T	G	missense	MEI1	4.63E-04	2.25E-08	6.59E-03	3.70E-10

Table S5 | Comparison to 10 genes associated with adult height in GIANT. In UK Biobank, FLNB (pLoF, missense|LC), NOX4 (missense|LC), OSGIN1 (missense|LC), and UGGT2 (pLoF) reach our genome-wide significance threshold (SKAT-O $p < 2.5 \times 10^{-7}$; Burden $p < 6.7 \times 10^{-7}$) (blue), but all are found nominally significant for either pLoF or missense variants (light blue). Related to STAR Methods.

Gene	UK Biobank			GIANT P-value			
	Annotation	Burden Test	SKAT-O	SKAT-Broad	VT-Broad	SKAT-Strict	VT-Strict
B4GALNT3	missense|LC	5.49E-03	6.64E-03	2.40E-05	1.90E-05	1.80E-05	**3.10E-07**
	pLoF	3.25E-06	4.76E-06				
	synonymous	6.46E-01	2.33E-01				
CCDC3	missense|LC	3.80E-02	9.03E-03	6.30E-04	6.30E-06	3.00E-07	**5.40E-09**
	pLoF	7.55E-01	6.25E-01				
	synonymous	4.00E-01	5.62E-01				
CRISPLD1	missense|LC	8.61E-02	1.37E-01	2.20E-07	**6.70E-11**	8.50E-06	8.90E-07
	pLoF	5.00E-03	6.84E-03				
	synonymous	3.81E-02	6.55E-02				
CSAD	missense|LC	3.57E-03	6.54E-03	2.30E-08	**2.40E-09**	0.83	0.59
	pLoF	3.33E-01	4.63E-01				
	synonymous	8.84E-02	6.97E-04				
FLNB	missense|LC	2.99E-08	2.12E-08	2.20E-06	5.10E-04	**2.40E-09**	3.20E-06
	pLoF	5.51E-11	9.35E-11				
	synonymous	7.37E-01	3.00E-02				
G6PC	missense|LC	5.77E-01	6.64E-02	1.30E-05	**3.60E-08**	5.50E-06	1.30E-06
	pLoF	3.03E-03	5.28E-03				
	synonymous	4.82E-01	4.06E-01				
NOX4	missense|LC	1.47E-10	5.27E-14	5.10E-06	**1.40E-07**	NA	NA
	pLoF	3.01E-04	5.04E-04				
	synonymous	8.31E-01	2.39E-01				
OSGIN1	missense|LC	8.14E-04	9.28E-11	**4.30E-11**	4.50E-05	0.19	0.18
	pLoF	7.40E-01	7.76E-02				
	synonymous	4.65E-01	6.53E-01				
SNED1	missense|LC	3.67E-02	6.24E-02	1.90E-05	**4.30E-09**	NA	NA
	pLoF	NA	3.69E-01				
	synonymous	1.32E-01	2.16E-01				
UGGT2	missense|LC	4.47E-05	1.09E-04	3.00E-05	**2.60E-07**	2.30E-05	4.80E-07
	pLoF	7.84E-09	6.51E-09				

	synonymous	8.76E-02	1.48E-01				

Table S6 | Comparison of 20 associations between missense variants and 7 major red blood cell phenotypes discovered at the genome-wide significant loci of the marginal tests in TOPMed; in UK Biobank, 9 of these associations are significant at $p < 8 \times 10^{-9}$ (blue), and 19 are found significant at $p < 0.05$ (light blue). Related to STAR Methods.

Phenotype	UKB phenocode	Locus	Allele (Ref)	Allele (Alt)	Gene	Annotation	P-value	
							TOPMed	UK Biobank
hematocrit (HCT)	30030: hematocrit percentage	chr6:26092913	G	A	HFE	missense	6.40E-17	5.05E-174
		chr22:37066896	A	G	TMPRSS6	missense	1.03E-26	3.28E-182
		chrX:154536002	C	T	G6PD	missense	3.36E-22	1.61E-03
hemoglobin (HGB)	30020: hemoglobin concentration	chr6:26092913	G	A	HFE	missense	2.16E-30	1.00E-300
		chr22:37066896	A	G	TMPRSS6	missense	3.16E-51	1.00E-300
		chrX:154536002	C	T	G6PD	missense	1.47E-28	2.02E-02
mean corpuscular hemoglobin (MCH)	30050: Mean corpuscular hemoglobin	chr11:5227003	C	T	HBB	missense	1.24E-23	2.06E-02
		chrX:154536002	C	T	G6PD	missense	2.12E-48	7.91E-03
mean corpuscular hemoglobin concentration (MCHC)	30060: Mean corpuscular hemoglobin concentration	chr6:26092913	G	A	HFE	missense	9.52E-17	3.59E-246
		chr11:5227003	C	T	HBB	missense	4.29E-43	2.37E-02
		chr22:37066896	A	G	TMPRSS6	missense	3.25E-26	5.70E-189
mean corpuscular volume (MCV)	30040: mean corpuscular volume	chr1:247876149	C	T	TRIM58	missense	1.77E-16	1.33E-118
		chr11:5227003	C	T	HBB	missense	1.36E-64	2.87E-04
		chr16:67184472	T	C	EXOC3L1	missense / synonymous	2.13E-09	7.48E-29
		chrX:154536002	C	T	G6PD	missense	3.96E-82	5.87E-02
red blood cell count (RBC)	30010: red blood cell (erythrocyte) count	chr11:5227003	C	T	HBB	missense	2.44E-22	1.49E-02
		chrX:154536002	C	T	G6PD	missense	3.72E-82	1.27E-04
red blood cell width (RDW)	30070: red blood cell (erythrocyte) distribution width	chr6:26092913	G	A	HFE	missense	5.80E-15	1.00E-300
		chr11:5227003	C	T	HBB	missense	1.59E-10	1.51E-02
		chrX:154536002	C	T	G6PD	missense	8.27E-106	1.87E-04
